# An Integrated Pipeline for Annotation and Visualization of Metagenomic Contigs

**DOI:** 10.3389/fgene.2019.00999

**Published:** 2019-10-15

**Authors:** Xiaoli Dong, Marc Strous

**Affiliations:** Department of Geoscience, University of Calgary, Calgary, AB, Canada

**Keywords:** metagenomics, metaproteomics, bioinformatics, gene prediction, functional annotation, taxonomic classification, pathway prediction, visualization

## Abstract

Here, we describe MetaErg, a standalone and fully automated metagenome and metaproteome annotation pipeline. Annotation of metagenomes is challenging. First, metagenomes contain sequence data of many organisms from all domains of life. Second, many of these are from understudied lineages, encoding genes with low similarity to experimentally validated reference genes. Third, assembly and binning are not perfect, sometimes resulting in artifactual hybrid contigs or genomes. To address these challenges, MetaErg provides graphical summaries of annotation outcomes, both for the complete metagenome and for individual metagenome-assembled genomes (MAGs). It performs a comprehensive annotation of each gene, including taxonomic classification, enabling functional inferences despite low similarity to reference genes, as well as detection of potential assembly or binning artifacts. When provided with metaproteome information, it visualizes gene and pathway activity using sequencing coverage and proteomic spectral counts, respectively. For visualization, MetaErg provides an HTML interface, bringing all annotation results together, and producing sortable and searchable tables, collapsible trees, and other graphic representations enabling intuitive navigation of complex data. MetaErg, implemented in Perl, HTML, and JavaScript, is a fully open source application, distributed under Academic Free License at https://github.com/xiaoli-dong/metaerg. MetaErg is also available as a docker image at https://hub.docker.com/r/xiaolidong/docker-metaerg.

## Introduction

Genome annotation is, literally, the annotation of features on assembled DNA molecules. Such features are, in the first place, genes, including those encoding proteins [“open reading frames” (ORFs)] and those encoding ribosomal or transfer RNA molecules. Annotation consists of the identification of such features and providing each feature with a meaningful list of hints about its possible biological function. Annotation is usually the final step of the automated computational processing of genomic or metagenomic data and the beginning of biology.

Depending on their background and research question, biologists will have different annotation needs. For example, when the research targets a single microbe, detailed gene-by-gene annotation of its genome would be desired. On the other hand, when the research targets a complete ecosystem, a high level summary of the functional potential of the associated metagenome might be the aim. These examples also display a different starting point for annotation. In the first case, it may consist of a single, near-perfect whole genome sequence. In the second case, it may consist of many MAGs of varying quality, unbinned metagenomic contigs, or even billions of unassembled reads.

What sets annotation apart from other computational steps in processing metagenomic data is that no benchmarks for annotation tools exist. That means that ranking these tools and objectively declaring a winner is not straightforward. The choice of the best annotation pipeline will depend on the data, the research question, the computational resources available, and the background of the researcher who needs to make sense of the annotation software’s hints and the way they are presented.

In practice, options for genome annotation come in two flavors: online platforms and standalone pipelines. Examples of online platforms are IMG (Chen et al., 2017), MG-RAST (Keegan et al., 2016), MicroScope (Vallenet et al., 2017), Mgnify (Mitchell et al., 2018), and Edge (Li et al., 2017). When opting for a platform, you avoid the need for local computational infrastructure or tedious installation and updating of tools and databases, while benefiting from online collaboration abilities. The platform may provide accession numbers for sharing data after publication, as these platforms may also be data repositories.

However, a platform might not offer the type of annotation needed for a specific research question or might be slower in the uptake of the latest selection of tools and databases. If such factors are important, opting for a standalone pipeline might be the way to go. Scientists who are fluent in scripting languages, such as Python or Perl, might even create their own pipeline from scratch. Examples of available standalone pipelines for annotation of assembled contigs, scaffolds, or whole genome sequences are Prokka (Seemann, 2014), DFAST-core (Tanizawa et al., 2018), and PGAP (Tatusova et al., 2016). Prokka is a very fast genome annotation pipeline. Its core concept is that some databases or tools provide better or more information than others. Once a gene is annotated with a positive “hit” to a good database, there is no need to perform additional searches. DFAST adds to this approach by using a faster similarity search tool (ghostx). It infers orthology assignments based on reciprocal-best-blast-hits between the query genome and a larger set of reference genomes, potentially including user-added custom reference genomes. It is thus especially useful to transfer annotations from a well-annotated reference genome. PGAP is used by the NCBI to annotate submitted whole genome sequences. It combines sophisticated gene prediction algorithms with gene assignments to its set of prokaryotic protein clusters (Klimke et al., 2009). As an institutional “gold standard” annotation, it emphasizes annotation standards and conventions, quality control, and due diligence during execution.

Here, we present MetaErg, an extendable standalone annotation pipeline developed for metagenome-assembled genomes (MAGs). Genome-centric metagenome data provides three major challenges. The first is that assembly quality can be relatively poor, and some contamination of MAGs with “foreign” genes can be expected. This challenge is addressed by performing fast similarity searches against a much larger database than would be needed to simply infer functions, to classify each gene taxonomically. This enables detection of potentially artefactual, hybrid bins or contigs. The second is that the user will likely need to make sense of many annotated genomes simultaneously. This challenge is addressed by visualizing and summarizing data, to enable quick inferences about encoded biological functions and pathways. The third is that, for many environmental microorganisms, meaningful/close reference genomes are not yet available. This challenge is addressed by always providing comprehensive information about each gene, derived from different tools and databases, to assign functions as well as practically possible for genes with low similarity to reference genes.

## Materials and Methods

### Program Implementation Overview

**Figure 1:**
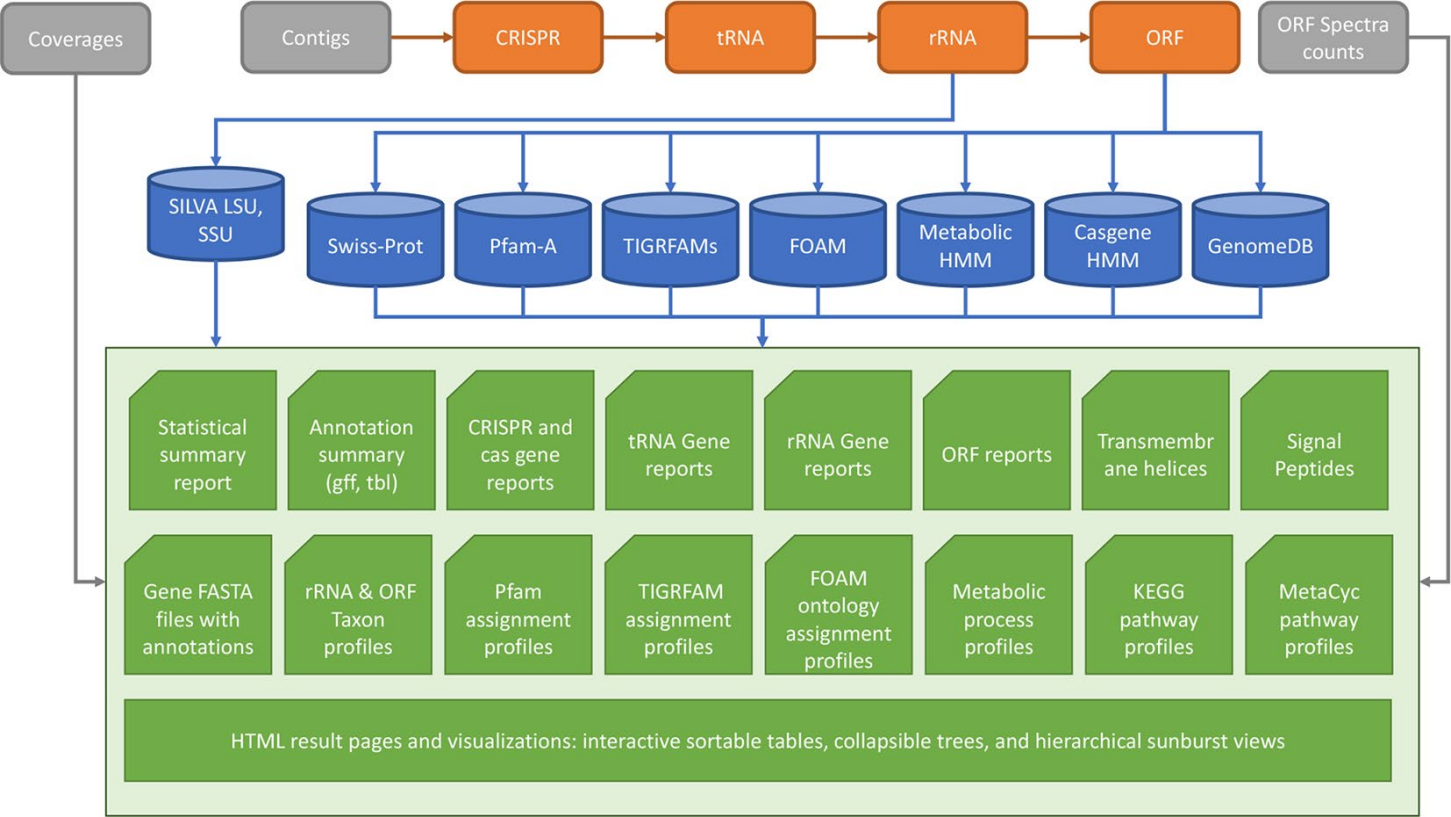
MetaErg annotation workflow. The input file to MetaErg is a FASTA file that contains the assembled contigs.

MetaErg is an integrated and fully automated pipeline for annotating metagenome-assembled contigs. It integrates a number of open-source tools and its modular design allows for flexible workflows, addition of new functions, and easy refactoring. MetaErg’s implementation consists of five main modules (**Figure 1**), including a command-line interface, an input data preprocessing module for filtering and formatting input DNA sequences, a structural annotation module for predicting biological features and elements, a function annotation module for inferring gene functions and classifying rRNA genes and ORFs to taxonomic lineages, and a presentation module for presenting annotation results in various summary reports and for visualization using HTML and JavaScript.

### Command Line Interface

MetaErg is a command line program, designed to run on a Linux server or cluster. It accepts a preassembled FASTA format DNA sequence file as the minimum required input. The default values for the optional parameters in the pipeline are optimized for metagenome analysis. Through a command-line interface, experienced users can interact with the program to customize the gene prediction and database searching parameters, enable or disable certain tools and functions, setup data filtering thresholds, and specify an output directory.

### Sequence Data Preprocessing

Every input DNA sequence is inspected, validated, and reformatted before annotation. The sequence identifiers in the input file must be unique; otherwise, the input file will be rejected, and the annotation process will be terminated. Any ambiguous nucleotides in the input sequence file are replaced by N. Gaps (-) and pads (*) are removed. Sequences shorter than a user defined minimum length are removed.

### Structural Annotation

MetaErg begins biological feature and element prediction by identifying CRISPR elements and noncoding RNA genes (tRNA, rRNA genes). Next, to avoid identification of artefactual protein coding genes overlapping with detected noncoding features, MetaErg masks these features by replacing them with Ns. Next, protein encoding genes are predicted. **Figure 1** shows the MetaErg workflow.

The identification of CRISPR elements is achieved using MinCED (Skennerton, 2016) with default parameters. tRNA genes are predicted with the ARAGORN program (Laslett and Canback, 2004).

Ribosomal RNA genes (5S, 5.8S, 16S, 18S, 23S, 28S) are identified and classified using rRNAFinder, an in-house tool package, which is included in the MetaErg release. rRNAFinder uses nhmmer (Wheeler and Eddy, 2013) to query locally built rRNA HMM profiles against the input contig sequences for detecting rRNA genes on the contigs. To build the rRNA HMM profiles, the “rfam.seed.gz” file was downloaded from the Rfam database (Kalvari et al., 2018). The FASTA-formatted rRNA gene alignments were extracted and written to separate files for each of the three domains of life (*Bacteria, Archaea, Eukaryota*), respectively. The alignment files for each domain were then used by the hmmbuild program in HMMER (Eddy, 2011) to create an rRNA gene HMM profile for the domain. Because a metagenome may contain rRNA sequences from all domains of life, in “metagenome” mode, rRNAFinder uses HMM models from all three domains of life. When multiple models yield hits to the same region, rRNAFinder outputs only the result of the model with the lowest *E*-value. When the *E*-value is the same for multiple hits, all best scoring predictions are kept. rRNAFinder uses blastn (Altschul et al., 1990) for classification of detected rRNA genes using the full-length SILVA SSU and LSU database (Quast et al., 2012). The standalone rRNAFinder tool is also freely available at https://github.com/xiaoli-dong/rRNAFinder.

Protein coding genes (ORFs) are predicted using Prodigal (Hyatt et al., 2010). ORFs shorter than 180 nucleotides are excluded from further analysis by default.

### Functional and Taxonomic Annotation

Metagenome functional annotation is very similar to genomic annotation and relies on comparisons of predicted genes with existing, previously annotated sequences. The goal is to propagate accurate annotations to correctly identified orthologs (Kunin et al., 2008).

Firstly, predicted ORFs are run through motif prediction tools. SignalP 5.0 (Armenteros et al., 2019) is run on all ORFs to predict the presence and absence of signal peptides and the location of their cleavage sites within an ORF. TMHMM (Krogh et al., 2001) is run on all ORFs to detect the transmembrane helices.

MetaErg uses profile HMMs and blast-based searches to detect similarity. All ORFs are searched against different databases. All search results are combined to associate query genes with functional categories, protein domains, KEGG Orthology (KO) terms, Gene Ontology (GO) terms, Enzyme Commission (EC) numbers, and metabolic potentials and traits. In brief, ORFs are searched with HMMs from Pfam-A (Finn et al., 2014), TIGRFAM (Haft et al., 2013), FOAM (Prestat et al., 2014), Metabolic-hmm (Anantharaman et al., 2016), and casgenes.hmm (Burstein et al., 2016) using the hmmsearch tool. ORFs are also searched against the SwissProt (BBairoch and Apweiler, 2000) database using DIAMOND (Buchfink et al., 2014). ORFs without any search outcomes are annotated as “hypothetical protein”.

MinPath (Minimal set of Pathways) was used to reconstruct metabolic pathways. MinPath minimizes parsimony and yields a conservative estimate of the biological pathways present in a query dataset (Ye and Doak, 2009). MetaErg uses MinPath to predict KEGG (Kanehisa and Goto, 2000) and MetaCyc (Karp et al., 2002) pathways. For predicting the minimal set of KEGG pathways that still explains the presence of the detected functional genes, an ORF-identifier-to-KO-number-mapping-file is provided as the input to MinPath. For inferring the list of MetaCyc pathways, an ORF-identifier-to-EC-number-mapping-file is provided as the input to MinPath. The mapping files are derived from the blast searches of the ORFs against the Swiss-Prot databases, as well as HMM searches against the FOAM and the TIGRFAMs database.

MetaErg classifies all ORFs based on best DIAMOND hits against a custom database, GenomeDB. To build GenomeDB, the Genome Taxonomy Database (GTDB) gtdbtk_r89_data.tar.gz (Parks et al., 2018) was downloaded from https://data.ace.uq.edu.au/public/gtdb/data/releases/release89/89.0/. Each genome included in GTDB was checked for presence in the NCBI RefSeq database. If present, the FASTA-formatted protein files were downloaded. Otherwise, the ORFs for the genome were predicted using Prodigal. The downloaded and locally predicted ORFs inherited their taxonomy from GTDB. To the GTDB data, only associated with *Bacteria* and *Archaea*, we added *Eukaryota* and viral data, by downloading the available NCBI RefSeq protein sequences of unicellular protozoa, fungi, plants (excluding *Embryophyta*), and viruses. The taxonomy of those proteins in GenomeDB was inherited from the NCBI records. For that, we inspected the assembly_summary.txt file, present in each NCBI RefSeq subdirectory (ftp://ftp.ncbi.nlm.nih.gov/genomes/refseq/), which associates each assembled genome with a “ftp_path” and a “species_taxid”. We retrieved the protein sequences of each available Eukaryote or viral genome by following “ftp_path”. The taxonomy of the protein sequences was obtained *via* “species_taxid”. This process was automated in a Perl script, enabling periodical updating of the database.

With a user-supplied coverage file generated by mapping reads from each sample to the assembled contig sequences, MetaErg quantifies the relative abundance of organisms, functions, metabolic processes, and pathways in each sample by tracking the number of reads that map to each gene family or orthologous group. The coverage file, generated using “jgi_summarize_bam_contig_depths” from MetaBat (Kang et al., 2015), is a tab delimited text file and the example coverage file is available at https://github.com/xiaoli-dong/metaerg/blob/master/example/demo.depth.txt. With a user-supplied metaproteomics spectral count file, MetaErg quantifies the abundance (in the proteome) of each taxon, function, metabolic process, and pathway based on expressed genes included in the spectral counts file for each sample. The spectral count file is a tab-delimited text file. The first column of the file is the gene id and all the columns after are the normalized protein expression level. The example metaproteomics spectral count file is available at https://github.com/xiaoli-dong/metaerg/blob/master/example/demo.plevel.txt.

### Output and Visualization

MetaErg reports annotations at the individual gene, single genome, and community level. For each gene, it reports the taxonomic classification and functional annotations, GO terms, EC numbers, KO terms, and its association with a metabolic pathway. At the community or genome level, MetaErg presents the taxonomic composition, protein function profiles, metabolic process profiles, and metabolic pathway profiles. A MetaErg output demo page is available at https://xiaoli-dong.github.io/metaerg/

**Figure 2:**
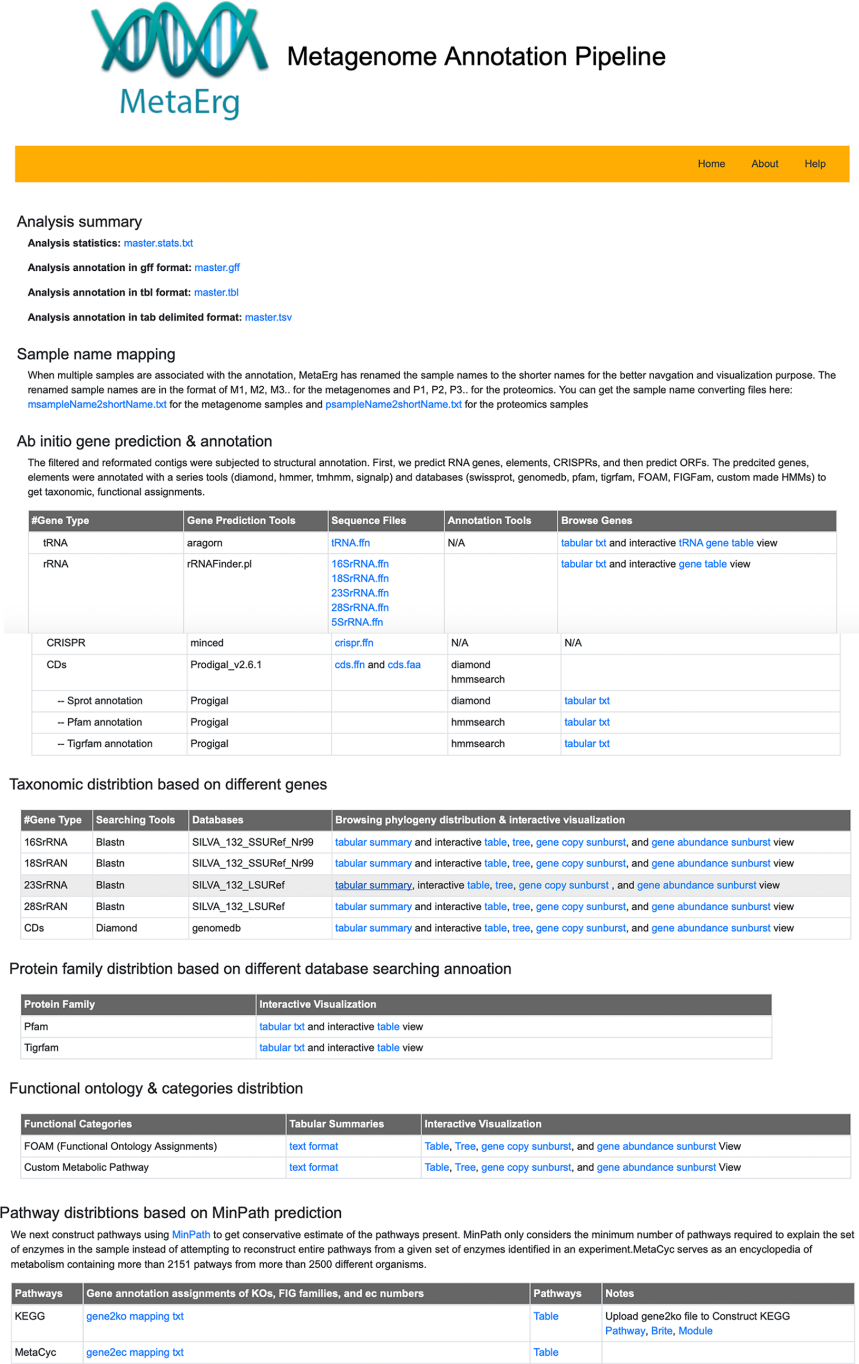
MetaErg HTML result page visually links extensive analysis text summaries, result data files, and accompanying visualizations together.

**Figure 3:**
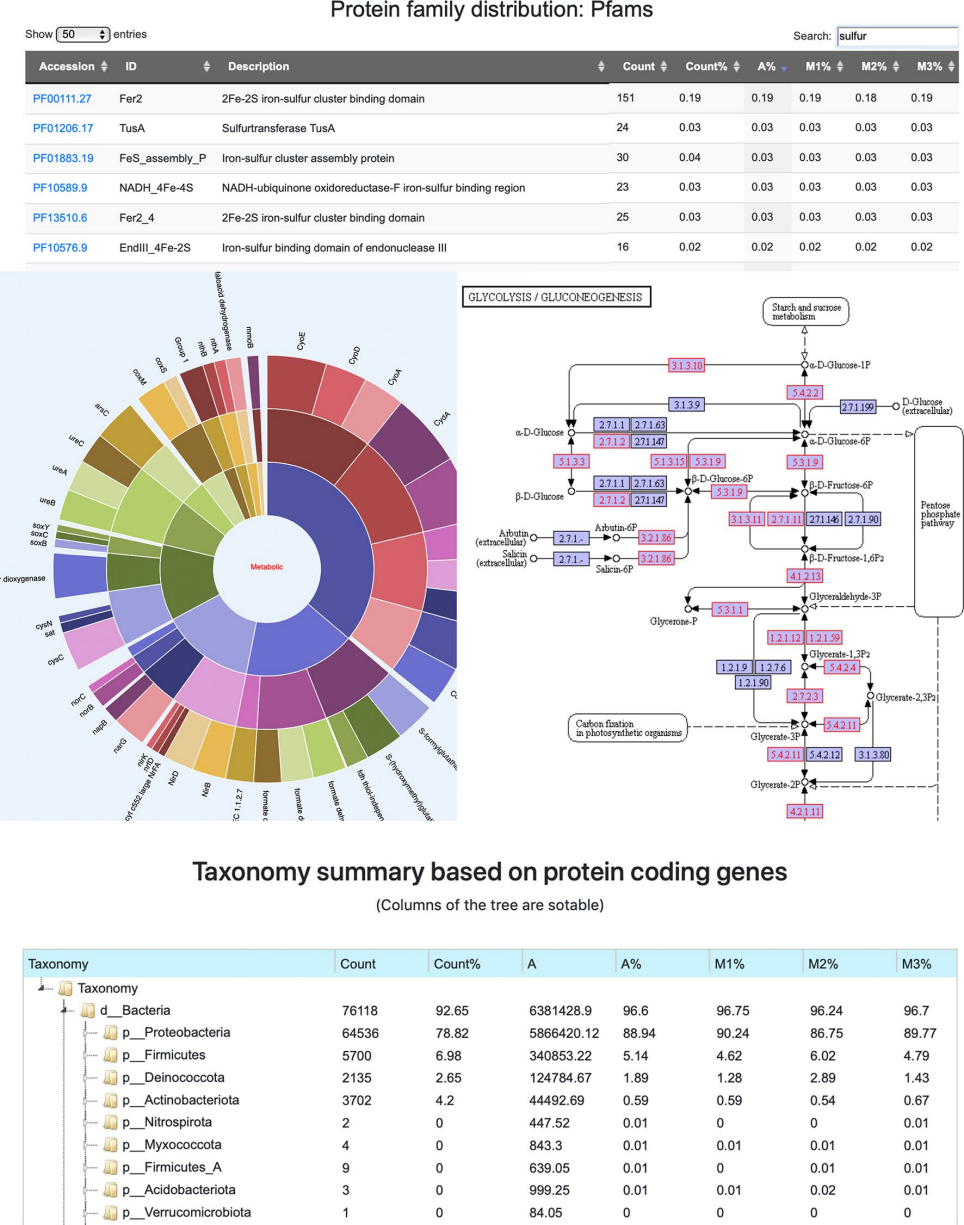
A screenshot montage of MetaErg output showing an example of the interactive Pfam annotation profile table, a hierarchical metabolic process sunburst view, a taxonomic summary tree view, and a KEGG pathway map. In the KEGG pathway map, the KOs presented in the analyzed dataset were highlighted.

To facilitate the exploration of complex metagenome annotation results and make sense of the data, MetaErg’s annotation reports are presented in various formats. The HTML result page (**Figure 2**) visually brings together text summaries, output data files, and accompanying visualizations. The interactive sortable and searchable gene, function, and profile tables, collapsible trees, sunburst hierarchical views of taxonomy and functional ontology, and other graphical representations, enable the effective interactive exploration, analysis, filtering, and intuitive navigation of complex metagenomic data (**Figure 3**).

The intermediate results, including those from feature predictions and similarity searches, are stored as files, which could be used to dig deeper into the data and validate the results later on. With the intermediate files in place, MetaErg will skip the steps used to generate them when the program is restarted with the same input parameters. This can greatly reduce the computational time when redoing the analysis.

### Generation of the Test Dataset

The paired-end Illunima raw reads of three biological replicates of a mock community sample (Kleiner et al., 2017, NCBI SRA accession numbers ERR1877474, ERR1877474, and ERR1877476) were filtered using BBDuk from the BBTools suite (Bushnell, 2014). Briefly, each read was screened by reference and by kmer for Illumina adapters (options: tbo tpe k = 23 mink = 11 hdist = 1 ktrim = r) and for Phix (options: k = 31 hdist = 1) and quality trimmed and filtered (options: qtrim = rl trimq = 15 minlength = 30 entropy = 0.5). After cleaning, the remaining reads were merged using BBMerge with default settings. The resulting merged single-end reads and unmerged paired-end reads from three samples were co-assembled together using metaSpades (Nurk et al., 2017) with default settings. After assembling, contigs shorter than 500 bp were excluded from further analysis.

Mapping of the quality-controlled reads from all three libraries back to the assembled contigs was preformed using BBMap with default settings. The depth coverage file “depth.txt” was generated using “jgi_summarize_bam_contig_depths” from MetaBat.

## Results

To test MetaErg and determine the computational footprint, a MetaErg job was submitted to a Linux cluster node (56 threads, 256 GB RAM) with the assembled contigs from a mock community as the input. The mock community consisted of 25 species of *Bacteria*, 1 *Archaeon*, 1 *Eukaryote*, and 5 *phages* (Kleiner et al., 2017). Assembly with MetaSpades resulted in 4,576 contigs (N50 126,358 base pairs, 85,113,339 base pairs total). The MetaErg job took 2.12 h to complete. The total CPU time needed was 50.5 h. When prediction of signal peptides and transmembrane helixes was included (with options “–sp –tm”), the run time and CPU time increased to 3.7 and 56.2 h, respectively. The average memory usage was 3 GB with peaks up to 9.5 GB. The total disk space used for the analysis including the intermediate files was 6.1 GB and the total disk space used for the final results was 482 MB.

The overall metagenome annotation predicted 20 CRISPR arrays, 878 tRNA genes, 70 rRNA genes (16S, 18S, 23S, 28S, 5S, 5.8S rRNA genes), and 80,407 ORFs. Of these, 48,723, 68,578, 22,001, 25,184, 475, and 437 ORFs were annotated with SwissProt, Pfam, TIGRFAM, FOAM, metabolic hmm, and casgene.hmm databases, respectively. Signal peptides were predicted for 1,480 ORFs and transmembrane helices were predicted for 18,766 ORFs. The relative abundances of taxa, functions, and pathways were nearly identical across all three biological replicates of the mock community.

**Figure 4:**
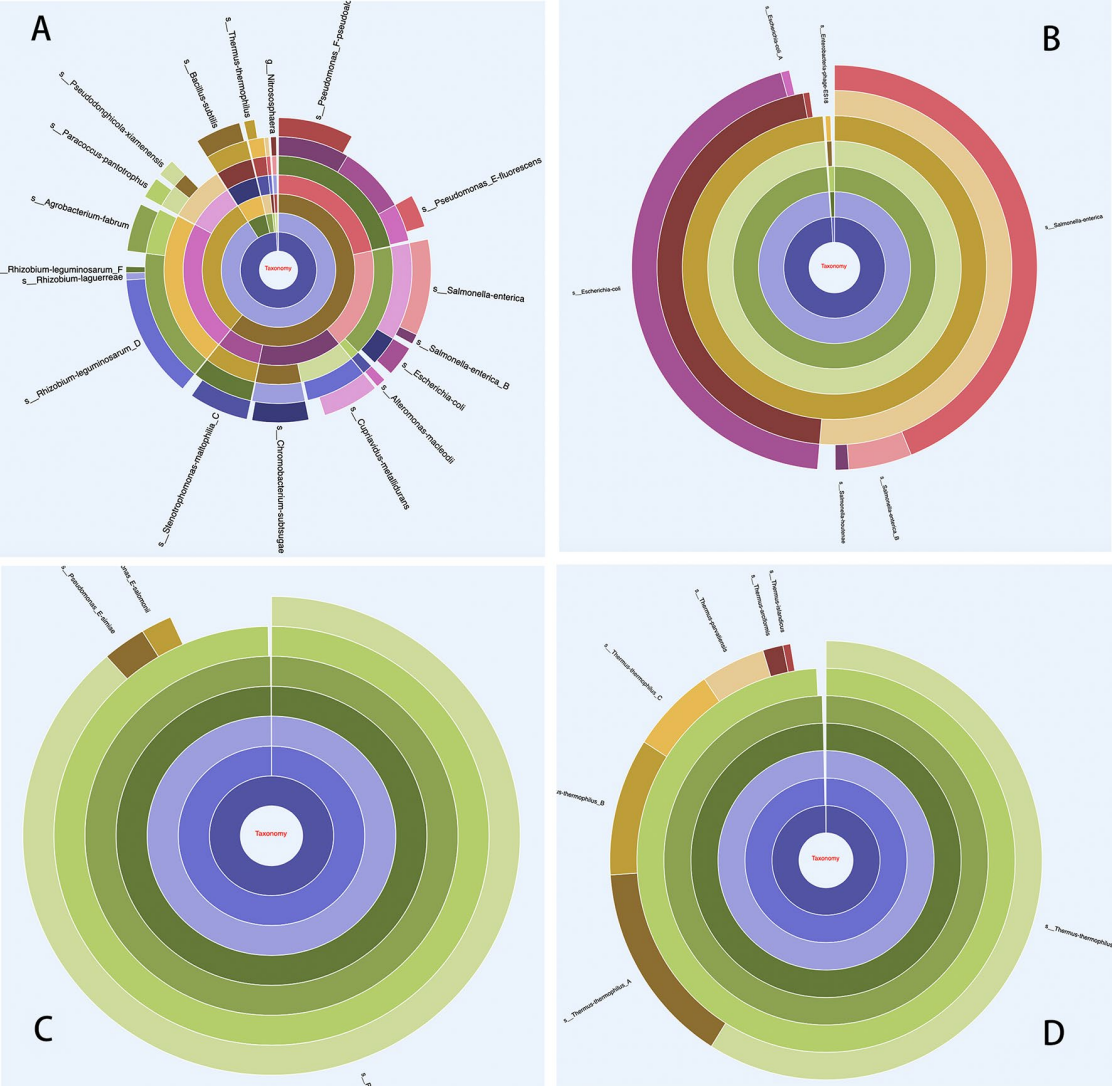
Taxonomy in hierarchical sunburst view. Each taxonomic rank is represented by one ring with the innermost circle representing the kingdom. From the inner to outer rings, the rings represent kingdom, phylum, class, order, family, genus, and species. The segmented areas on the ring are proportional to the relative abundance of the taxon. **(A)** Overall taxonomic distribution profile from all ORFs, which provides insight into the community taxonomic distribution as a whole; **(B)** An example of chimeric MAG, displaying contamination, and this MAG was 99.42% complete and 97.14% contaminated, as assessed by CheckM. The taxon classification profile was based on ORF taxonomic assignment from the MAG; **(C)** and **(D)** Examples of uncontaminated MAGs.

MetaBat binning of the contigs with default parameters produced 14 useful MAGs (>70% completeness, <5% contamination). MetaBat binned relatively few MAGs for this dataset, because the three available read sets were from replicate samples and were not useful for differential coverage based binning. The annotations for each MAG were extracted directly from the overall annotations using MetaErg’s utility scripts. The phylogenetic affiliations of MAGs were estimated according to the taxon assignments of ORFs and rRNA genes in the MAGs and visualized in the interactive HTML trees and sunburst hierarchical views. The HTML visualizations can help users visually validate the binning outcomes and identify chimeric MAGs or contamination with genes from other community members (**Figure 4**). Each gene from each MAG was assigned comprehensive information derived from different resources with different tools (**Table 1**).

**Table 1:** An example showing information associated with each protein coding gene after MetaErg analysis.

TAG	Value
ID	mockEvenCell|17112
contigid	NODE_27_length_371703_cov_24.485093
allec_ids	7.1.1.-; 1.8.4.8
allko_ids	K00390; K00338;
allko_ontology	L1:18_Sulfur compounds metabolism;L2:Sulfur compounds cycle;L3:Sulfate reduction (assimilatory);L4:;
depth	82.0316;
foam_ecs	1.8.4.8;
foam_kos	K00390;
foam_target	db:FOAM-hmm_rel1a.hmm|HMMsoil748 63 117 evalue:2.5e-13 qcov:30.55 identity:40.00 score:41.9 seqT:47.9 name:KO:K00390_1.8.4.8;
genomedb_oc	d__Bacteria;p__Proteobacteria;c__Gammaproteobacteria;o__Betaproteobacteriales;f__Burkholderiaceae;g__Cupriavidus;
genomedb_target	db:genomedb|GCA_900185755.1|FYAX01000037.1_317 1 163 evalue:1.4e-89 qcov:100.00 identity:100.00;
pfam_desc	4Fe-4S binding domain;
pfam_id	Fer4;
pfam_target	db:Pfam-A.hmm|PF00037.27 61 80 evalue:2e-07 qcov:12.22 identity:55.00 score:24.1 seqT:53.6 name:Fer4; db:Pfam-A.hmm|PF00037.27 97 118 evalue:5.5e-11 qcov:13.44 identity:63.64 score:35.4 seqT:53.6 name:Fer4;
sport_desc	NADH-quinone oxidoreductase subunit I;
sprot_ec	7.1.1.-;
sport_go	GO:0005886;GO:0051539;GO:0005506;GO:0050136;GO:0048038;
sport_kos	K00338;
sport_target	db:uniprot_sprot|sp|Q1LPV5|NUOI_CUPMC 1 163 evalue:4.1e-65 qcov:100.00 identity:100.00;
tigrfam_go	GO:0050136;GO:0055114;
tigrfam_desc	NADH-quinone oxidoreductase, chain I;
tigrfam_id	NuoI;
tigrfam_mainrole	Energy metabolism;
tigrfam_sub1role	Electron transport;
tigrfam_target	db:TIGRFAMs.hmm|TIGR01971 20 141 evalue:2.1e-48 qcov:73.93 identity:52.46 score:152.8 seqT:153.0 name:TIGR01971;

## Discussion

With MetaErg, we provide a standalone and fully automated metagenome and metaproteome annotation pipeline. Compared to other standalone annotation pipelines, such as Prokka (Seemann, 2014) and DFAST-core (Tanizawa et al., 2018), MetaErg requires much more time to run and requires more computational resources. However, these extra resources result in more comprehensive annotation and visualization. Taxonomic classification of each gene, provided by MetaErg, enables detection of potential assembly or binning artifacts, as shown in **Figure 4**. More comprehensive annotation enables better inferences about gene function for genes that are more dissimilar to validated reference genes. High level visualization of pathways, and integration of expression data, enables more effective navigation of the full complexity of a metagenome. Thus, MetaErg provides solutions to challenges specific to metagenomes, which come at a computational cost.

Annotations are generated and visualized for the complete metagenome, as well as for each individual MAG. Depending on the research question, users can opt to only annotate a few selected MAGs. Alternatively, they could annotate the entire metagenome first and then use one of MetaErg’s utility scripts to extract annotations for each individual MAG. While the annotation of the complete metagenome provides insight into a community’s taxonomic composition and metabolic potential, analysis of an individual MAG presents this information for a single organism or population.

Because of the size and density of information in metagenome analysis, exploration of the data presents an overwhelming task that often takes many years to complete (Devlin et al., 2018). To address that challenge, MetaErg produces annotation summary results in various formats. The interactive HTML interface brings all annotation results together in sortable and searchable tables, collapsible trees, and other graphic representations, enabling intuitive navigation of complex data.

With typically massive metagenomic data, similarity-based functional analysis approaches usually suffer from excessive computation time. To address that, DIAMOND is used instead of BLASTP. Diamond is 500 to 20,000 times faster than Blast search tools with a similar degree of sensitivity. To overcome the computational bottleneck and to speed up the functional annotation process, the most time-consuming steps such as database searching in MetaErg are parallelized. Therefore, they run effectively on multicore processors.

Due to the high diversity and large proportion of uncharacterized microbial taxa in most environmental habitats, many microorganisms from environmental samples have no close reference genomes available. While a blast-like tool can quickly identify very similar genes, more distantly related genes can be missed. A profile HMM-based strategy is better at finding more divergent matches and gains sensitivity by incorporating position-specific information into the alignment process and by quantifying variation at each sequence position (Skewes-Cox et al., 2014). MetaErg relies on both Blast and HMM databases (PFAM, TIGRAMs, Metabolic-hmm, casgenes.hmm, and FOAM). FOAM is a manually curated HMM database for identifying functional genes in environmental metagenomes and transcriptomes. Because FOAM was last updated in 2014, we are implementing the addition of UniRef as an alternative, for the next release of MetaErg. Gene annotations such as the EC number and KO number, currently provided by FOAM, could be retrieved from UniRef instead.

SignalP and TMHMM are established signal peptide and transmembrane helix prediction tools. Phobius (Kall et al., 2004) is a combined transmembrane topology and signal peptide predictor. Phobius runs faster on the same dataset than SignalP and TMHMM. However, running Phobius on a 64-bit Linux system requires manually changing its source code before running, due to problems with the included decodeanhmm program. For that reason, we did not select Phobius as a dependency for MetaErg.

Taxonomic classification of genes by similarity searches can be misleading because of the uneven representation of taxa in databases. This can lead to a bias towards highly sampled taxa (Kunin et al., 2008). In addition, with the growing size of the databases, searching all available sequence information becomes computationally challenging. To partially overcome this challenge and improve the classification of uncultured organisms, MetaErg classification databases were built based on GTDB, which provides a more even sampling across *Bacteria* and *Archaea*. Because microbial communities usually also comprise *Eukaryotes* and viruses, we have also added protein sequences of unicellular protozoa, fungi, plants (excluding *Embryophyta*), and viruses. Because MetaErg currently uses Prodigal for gene prediction, it is unable to correctly predict protein sequences of *Eukaryotes*. We are currently working on implementing workflows for better predictions of eukaryotic coding sequences, which will become part of the next version of MetaErg. Likewise, effective identification and analysis of viral contigs is currently still lacking and will become part of the next version.

Although advances in metagenomics have enabled a better understanding of microbial phylogenetic and functional gene compositions in microbiomes, it is also desirable to know which genes are actually expressed. This could be visualized based on transcriptomics or proteomics data (White et al., 2016). Currently, MetaErg enables visualization of expression based on proteomics data only. Visualization of transcriptomics data is planned for a future release.

In conclusion, MetaErg is an easy to use and robust metagenome analysis pipeline. It produces comprehensive analysis reports in various formats. The interactive visualizations help to ease the challenge in interpreting complex analysis results. MetaErg is fully open source and portable, available as a docker image, designed to run on moderately sized computational clusters. Its modular architecture enables addition of new functions. In the future, MetaErg will be expanded by adding new functionality focusing on identification and annotation of eukaryotic and viral MAGs, annotation and discovery of gene clusters encoding production of secondary metabolites, and visualization of transcriptomic data.

## Data Availability Statement

Publicly available datasets were analyzed in this study. This data can be found here: https://www.ncbi.nlm.nih.gov/bioproject/?term=prjeb19901.

## Author Contributions

MetaErg was conceived by XD and MS; XD implemented program with the input from MS; and XD and MS wrote the paper.

## Funding

We acknowledge financial support from the Natural Sciences and Engineering Research Council of Canada (NSERC), the Canada Foundation for Innovation (CFI), the Canada First Research Excellence Fund (CFREF), Alberta Innovates, the Government of Alberta, Genome Canada, the University of Calgary, and the International Microbiome Center.

## Conflict of Interest

The authors declare that the research was conducted in the absence of any commercial or financial relationships that could be construed as a potential conflict of interest.
